# Esthetic improvement of a cutaneous sinus tract of odontogenic origin

**DOI:** 10.11604/pamj.2020.37.204.21596

**Published:** 2020-10-30

**Authors:** Yousra Zemmouri, Saliha Chbicheb

**Affiliations:** 1 Department of Oral Surgery, Consultation Center of Dental Treatment, Faculty of Dentistry, University Mohamed V, Rabat, Morocco

**Keywords:** Esthetic improvement, cutaneous sinus tract, odontogenic infection

## Abstract

A cutaneous sinus tract of dental origin is relatively uncommon and may easily be misdiagnosed because of its unusual occurrence and absence of dental symptoms. Extraoral drainage depends on the location of the affected tooth as well as on specific factors such as the virulence of the microorganism, resistance of the patient's body, and the relationship between anatomy and muscle facial attachments.

## Introduction

An odontogenic cutaneous sinus tract is a pathologic channel that initiates in the oral cavity and exits at the cutaneous surface of the face or neck. It might resemble an ulcer, cyst, furuncle, or retracted, sunken skin [1]. The purpose of this paper is to present a case of an odontogenic cutaneous sinus tract in the nasolabial fold, showing the etiology, the surgical management. Healing and esthetic improvement occurred only after surgery and removal of the causal tooth and the affected tissue. The aetiology of an extraoral sinus tract of odontogenic origin within the face and neck region has been cited as resulting primarily as result of caries and subsequent pulpal necrosis, secondarily as a consequence of trauma-related pulpal necrosis and rarely, as a result of a periodontal infection [2]. Without effective treatment, infection can spread from the necrotic pulp into the root apical area around the tooth, resulting in apical periodontitis. Seeking the path of least resistance, the purulent by-products of infection can then travel through the bone and the soft tissue [3]. The purpose of this paper is to present a case of an odontogenic cutaneous sinus tract associated with the mental protuberance region, showing the etiology, the surgical management. Healing and esthetic improvement occurred only after surgery and the root canal treatment.

## Patient and observation

**Figure 1:**
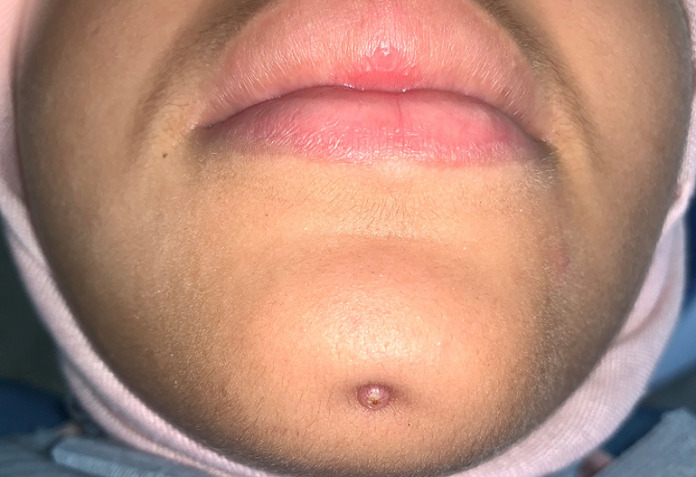
view showing a cutaneous sinus tract associated with the mental protuberance region

**Figure 2:**
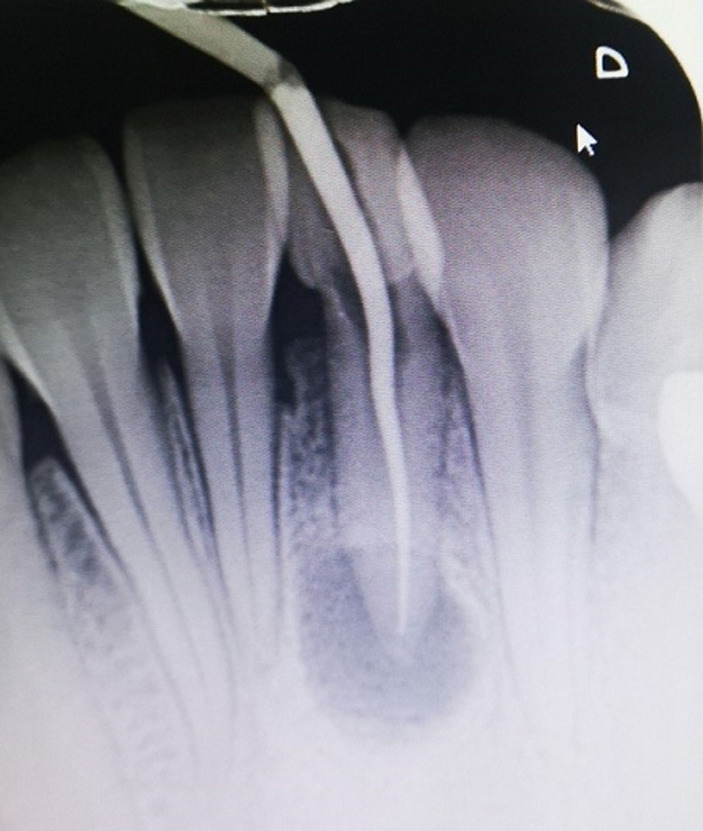
periapical radiograph showing an apical radiolucency, surrounding the apex of 31

A healthy 17-year-old female patient presented herself to the dental hospital of the Faculty of Rabat complaining of discomfort and nonesthetic appearance of her face. The patient presented an extraoral sinus tract associated with the mental protuberance region, with an approximate size of 5 mm x 5 mm. The nodule was soft with minimal purulent discharge. During the interview, the patient reported that the sinus tract had appeared 3 months before and several treatment attempts based on antibiotics and dermatologic ointment had been made after visiting a dermatologist without any improvement (Figure 1). No intraoral vestibular swelling was present, but a stalk-like communication was palpable and continuous from the apical area of tooth 13 to the cutaneous lesion. Periapical intraoral radiographs of the mandibular incisors showed radiolucencies associated with the apical area in both teeth 42 and 31. No intraoral vestibular swelling was present, but a stalk-like communication was palpable and continuous from the apical area of tooth 31 to the cutaneous lesion. Periapical radiograph shows the presence of an apical radiolucency of an approximate 0.5 mm diameter, more or less round, badly limited, of inhomogeneous color, surrounding the apex of 31 (Figure 2).

**Figure 3:**
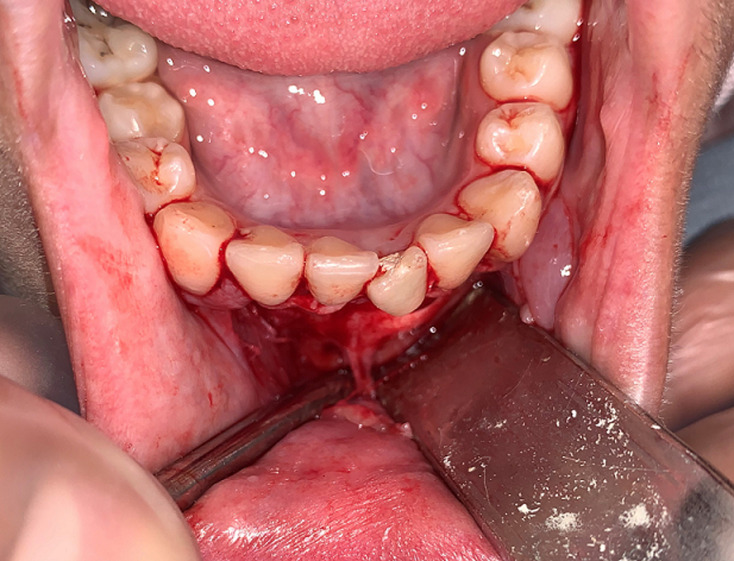
detachment of the cord-like tract from its alveolar origin

**Figure 4:**
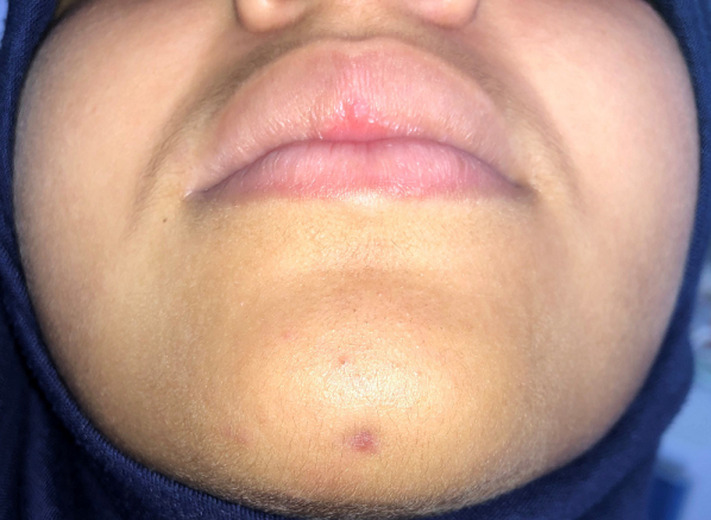
clinical appearance of the cutaneous sinus tract after 10 days showing the relaxation of the skin

A working diagnosis of an extraoral sinus tract related to a previously unknown traumatic injury to the incisors with consequent pulpal necrosis and periapical infection associated with the mandibular incisor tooth 31. Root canal treatment of tooth 31 commenced. The 31 was extirpated and dressed with a non-setting calcium hydroxide. Tooth 31 was then obturated with gutta-percha and hermetically sealed. The patient was monitored post-initial treatment at 2 months. The patient at 2 months review was concerned regarding the residual extraoral scarring affecting the mental protuberance region. For it we started the surgical procedure with the reflection of a full-thickness flap which allowed to observing that one of the fistulous tract extremities stuck to the bone around the apical region of 31. Consequently, the area was dissected to surgically remove the cord-like tract (Figure 3). After the excision, the right cheek immediately found back its normal appearance with the relaxation of the retracted skin. The patient was controlled 10 days after surgery; a relaxation was noted in the cheek with a beginning of the cutaneous sinus tract healing. After 2 months, there was an esthetic appearance improvement of the former lesion (Figure 4).

## Discussion

Cutaneous sinus tracts in the lower face is frequently described as an extraoral manifestation of pulpal-periradicular pathosis, might have other etiologies related to infectious disease such as mycobacterial infections, actinomycosis, deep fungal infections, osteomyelitis, or tumors such as pyogenic granulomas, epidermal cysts, squamous cell carcinomas, or congenital malformations such as thyroglossal duct cysts, brachial cleft cysts, and salivary gland fistulas [4]. Like our patient, affected patients usually search help from surgeons or dermatologists rather than dentists and often undergo multiple inappropriate treatments. Furthermore, a dental origin can present a diagnostic challenge because these tracts often have a clinical appearance similar to other uncommon facial lesions [5,6] The correct diagnoses allow prompt resolution of the lesion, while misdiagnoses can delay treatment and lead to facial deformity [7]. The pathophysiology of facial lesions of dental origin is well described in the literature. The infection from the root of the tooth spreads through the path of least resistance, perforating the outer cortex of the mandible or maxilla. The path in the soft tissues is decided by facial muscle attachments. Most sinuses in adults pass within the muscle attachments (the buccinator, the mylohyoid, and the masseter muscles); and this results in a sinus that opens intra-orally. In these cases, the diagnosis is easily made. In adults the path of the sinus is rarely above or below these muscle attachments; resulting in an extra- oral facial lesion or sinus [8]. Hence, there is greater likelihood for the sinus to go beyond the muscle attachment; presenting extra-orally as a facial lesion [9]. Intraoral and dental examinations are critical for making the diagnosis. In particular, the examiner should look for dental caries or restorations and periodontal disease. He should keep in mind that the involved tooth can even appear asymptomatic [10].

Radiographic findings are also important for the diagnosis and identification of affected teeth. Radiographic findings are also important for the diagnosis and identification of a carious tooth or retained roots along with the associated radiolucency periapical lesion, which may be a granuloma or a cyst [11]. Although conventional panoramic radiographs might be useful for identifying the location of suspected teeth, confirming which tooth is associated with a cutaneous draining sinus tract is difficult, especially when multiple teeth are suspected. Some studies have suggested inserting a probe or an endodontic gutta-percha point through the sinus tract to obtain radiographs so as to aid the identification of the affected teeth [6]. The treatment of odontogenic cutaneous sinus tracts requires eradication of the original source of infection by means of nonsurgical root canal treatment (if the tooth can be preserved) is most important for treatment of odontogenic cutaneous sinus tract sometimes complemented by surgery [12]. Once the tooth is treated, the need for surgical excision is controversial. Some studies have suggested complete excision of the sinus tract lining, while others have suggested that surgical treatment and antibiotic therapy are not necessary after dental treatment [13,14]. In this case, root canal treatment was sufficient for healing to occur. Plastic surgery may be needed at a later stage if healing results in cutaneous retraction. In our case report, the tooth was restorable and the root canal treatment was done and followed by the removal of the cord from its origin to the point of skin attachment, because at 2 months appointment fistula is always productive, which allowed relaxation of the facial skin, elimination of the skin dimpling in the affected area, and restoration of normal facial contours. Two months after treatment, the lesion on the chin had healed without recurrence and which allowed obtaining a normal esthetic appearance leaving only a slight hyperpigmented region.

## Conclusion

Facial lesions of dental origin are rare. This case highlights the significance of including odontogenic origins to the differential diagnosis of orofacial skin lesions. Early correct diagnosis, based on radiologic evidence of a periapical root infection, and treatment of these lesions can help prevent unnecessary and ineffective antibiotic therapy or surgical treatment, reducing the possibility of further complications.
